# Higher Rates of Untreated Distress in Older Versus Younger Adults With Indolent Lymphoma

**DOI:** 10.1093/geroni/igaa057.1510

**Published:** 2020-12-16

**Authors:** Kelly Trevino, Peter Martin, John Leonard

**Affiliations:** 1 Memorial Sloan Kettering Cancer Center, New York, New York, United States; 2 Iowa State University, Ames, Iowa, United States; 3 Weill Cornell Medicine, New York, United States

## Abstract

Indolent lymphomas are incurable but slow-growing cancers, resulting in a large number of older adults living with these diseases. Patients typically live with their illness for years with the knowledge that disease progression is likely. Yet, little is known about psychological distress in this population. This study examined rates of and the relationship between distress and mental health service use in older and younger adults with indolent lymphomas. Adult patients diagnosed with an indolent lymphoma (e.g., follicular lymphoma, marginal zone lymphoma) within the past six months completed self-report surveys of distress (Hospital Anxiety and Depression Scale; HADS) and mental health service use since the cancer diagnosis (yes/no). Descriptive statistics, t-tests, and chi-square analyses were used to examine study questions. The sample (n=84) included 35 patients 65 years or older. Across the entire sample, 21.4% screened positive for distress on the HADS; 58.8% of these patients did not receive mental health services. Older adults reported lower distress levels than younger adults (17.1% v. 24.5%; p=.038). Among younger adults, 50% of distressed patients received mental health services; only 20% of distressed older adults received mental health services. Distress was associated with mental health service use in younger adults (p=.004) but not in older adults (p=.17). Older adults with indolent lymphomas have higher levels of untreated distress than younger adults. Research on the mechanisms underlying these age differences (e.g., stigma toward mental health services, ageism) would inform interventions to increase rates of mental health service use and reduce care disparities due to age.

